# Influenza Vaccination Quality Improvement as a Model for COVID-19 Prophylaxis

**DOI:** 10.7759/cureus.12549

**Published:** 2021-01-07

**Authors:** Justin Chin, YaQun Zhou, Chijen L Chen, Christine M Lomiguen, Suzanne McClelland, Mary Lee-Wong

**Affiliations:** 1 Medicine, Lake Erie College of Osteopathic Medicine, Erie, USA; 2 Family Medicine, LifeLong Medical Care, Richmond, USA; 3 Primary Care, Touro College of Osteopathic Medicine, New York, USA; 4 Internal Medicine, Zhongshan School of Medicine at Sun Yat-sen University, Guangzhou, CHN; 5 Pathology, Lake Erie College of Osteopathic Medicine, Erie, USA; 6 Allergy and Immunology, Mount Sinai Beth Israel Medical Center, New York, USA

**Keywords:** covid-19, influenza vaccine, influenza, flu, vaccine, covid-19 vaccine, vaccine hesitancy, coronavirus disease, quality improvement, immunization

## Abstract

Introduction: Many comparisons have been made on the effect and impact of COVID-19 on influenza pandemics of history. Therefore, it is reasonable to infer that the strategies utilized by healthcare providers to improve influenza vaccination rates can similarly be applied to the administration of a COVID-19 vaccine. The purpose of this study was to determine the rationale of low influenza vaccination rates in an urban allergy clinic and how to improve patient education and knowledge regarding the importance of influenza vaccination. A three-year comparison of interventions is presented as well as its application to future COVID-19 vaccinations.

Methods: This study was performed at an outpatient allergy and clinical immunology practice (MSBI) with hospital affiliation in New York City, New York. A quality improvement medical committee was formed to optimize influenza vaccination rates to greater than 71% and established standardized protocols regarding patient intake workflows, vaccine counseling, and documentation. Patient records from four providers were used for this study to compare pre-and post-intervention rates.

Results: 984 patients met inclusion criteria, with a normal distribution of ages (18-80), race, and sex. Average vaccination rates prior to the intervention were 9.25-13.60%. The average vaccination rate after the intervention was 91.34%.

Discussion: The MSBI quality improvement study identified key areas to address in improving influenza vaccination rates. Vaccine hesitancy, public misinformation, and ambivalence surrounding vaccination with egg allergies or during a subcutaneous immunotherapy injection were all topics addressed during the 2018-2019 intervention year. Additional attention was also put toward provider education and standardization of documentation. Shared decision making and intensive education/outreach efforts are needed by physicians and patients alike to overcome vaccine hesitancy. In comparing this to upcoming COVID-19 vaccine challenges, similar barriers will likely also need to be addressed. Greater research is needed to understand patient motivations regarding hesitancy specific to the COVID-19 vaccine.

Conclusion: As evidenced in the yearly battle with influenza and now the COVID-19 pandemic, it has become essential to identify and implement multi-level strategies to maximize vaccination rates, especially amid a global pandemic. With COVID-19 vaccines reaching emergency approval stages, it is important for healthcare providers to start creating workflows and strategies to address patient inquiries. The influenza vaccination quality improvement project presented here can be used as a guideline for future evaluations of COVID-19 vaccination efforts.

## Introduction

Influenza, commonly known as the flu, is caused by the influenza virus, which is part of the *Orthomyxoviridae* family [1]. Three types (A-C) have been known to infect humans, with a fourth (D) having the potential to do so. Three to five million cases occur annually, with up to 500,000 deaths worldwide [2]. Symptoms range widely from mild to moderate respiratory and constitutional symptoms with fever, fatigue, and cough to more severe sequelae such as pneumonia and sepsis. Infants, the elderly, and those with other medical comorbidities have higher rates of complications requiring hospitalization [3]. Direct transmission, residual airborne aerosolization, and fomites are the common methods of transference [1,2]. Unlike the common cold and other viral infections, primary prevention in the form of a vaccine, first developed in the 1940s, has been shown to decrease influenza infections and associated complications [4]. Due to frequent mutations secondary to genetic shifts and drifts, the influenza vaccine is reformulated every year based on predicted high virulence strains [5]. Yearly campaigns by public health and medical providers alike attempt to encourage communities to receive the influenza vaccine in hope of mitigating disease spread and burden.

In comparison, Coronavirus Disease 19 (COVID-19) is caused by the severe acute respiratory syndrome coronavirus 2 (SARS-CoV-2) virus from the *Coronaviridae* family [6]. Initially tied to Wuhan, China, COVID-19 soon evolved into a global pandemic with rapid transmission rates and high mortality. With variations in both enforcement and public adherence to social distancing and other containment methods, over 1.3 million deaths worldwide have been attributed to COVID-19, with over 230,000 in the United States alone [7]. Over the past several months, numerous pharmaceutical companies have entered the race to develop a vaccine, with nine candidates entering phase III trials in October 2020; three of the nine pharmaceutical companies have begun the process to seek emergency Food and Drug Administration (FDA) approval for their vaccines in November 2020 [8,9]. Compared to previously developed vaccines, the timeline and regulations surrounding a COVID-19 vaccine have been truncated with intentions of fast-tracking a solution for the current pandemic that has crippled individual lives and global economies [10]. Recent studies for COVID-19 vaccination acceptance have detailed noticeable demographic and geographic disparities, particularly when compared to influenza vaccination acceptance [11]. Therefore, it is reasonable to infer that the strategies utilized by healthcare providers to improve influenza vaccination rates can similarly be applied to the administration of a COVID-19 vaccine.

The purpose was to determine the rationale of low influenza vaccination rates in an urban allergy clinic and how to improve patient education and knowledge regarding the importance of influenza vaccination. To do so, a quality improvement project was convened to improve rates of influenza immunization in which provider education and patient perceptions of vaccination with allergy sensitization were addressed. A three-year comparison of interventions is presented as well as its application to future COVID-19 vaccinations.

## Materials and methods

Setting

This study was performed at an outpatient allergy and clinical immunology practice (MSBI) with hospital affiliation in New York. A quality improvement medical committee was formed consisting of medical personnel, hospital representatives, and other pertinent stakeholders after multiple years of below-average influenza rates (less than 14% of eligible encounters). As noted annually by the Advisory Committee on Immunization Practices in conjunction with the Centers for Disease Control and Prevention, age-appropriate and non-medically contraindicated influenza vaccination is recommended for all groups greater than six months of age [1,4]. Utilizing measures outlined under the Physician Quality Reporting System (PQRS), several goals were outlined to improve patient care, one of which was to increase the rate of influenza immunization to 71% [12].

Intervention development and implementation

**Figure 1:**
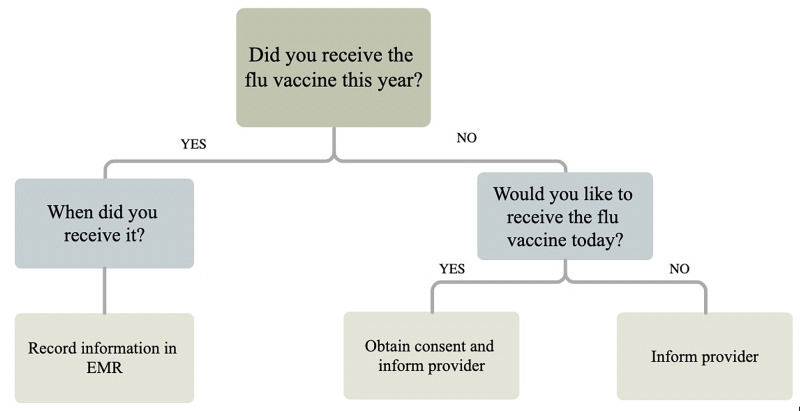
Schematic diagram of staff workflow intervention for increasing influenza vaccination rates.

An informal discussion amongst providers identified the following challenges, which included keeping influenza documentation metrics at the forefront of providers’ minds and reluctance to give vaccinations in conjunction with allergy desensitization due to potential challenges identifying the cause of adverse events. Similarly, patient anecdotes of vaccine hesitancy and media portrayal of vaccination were also reviewed. Interventions included education for the provider and support staff regarding evidence-based studies, presentation of adverse events vis-a-vis vaccination, and vaccine counseling. Prior to the 2019 influenza seasons, providers met to identify a vaccination goal (71%), established standardized protocols regarding vaccine counseling and documentation, and created a workflow that included staff asking about influenza vaccination during patient intake (Figure 1). Review and presentation of literature regarding vaccine hesitancy and common concerns regarding influenza vaccination in an allergy clinic setting were also discussed. 

During the influenza season, the medical staff asked each patient during intake if the patient had received the influenza vaccine. If patients had not received the influenza vaccine, but were interested, then the staff would obtain consent. If the patient had not received the vaccine and were not interested, then staff were instructed to inform the provider. Providers were tasked with discussing reasons for influenza vaccine refusal and encouraging vaccination as appropriate. Healthcare Common Procedure Coding System procedural codes (G8482 and G8483) were used by providers to ensure standard documentation for eligible visits and accurately reflect influenza vaccination counseling during the visit.

Data collection and analysis

Patient records from four providers were used for this study. Inclusion criteria were as follows: (1) Above the age of 18 to 80 years, (2) had at least one visit with one of the participating providers between October and March, and (3) did not have any contraindications to receiving the influenza vaccine. Performance reports were generated by an external reviewer to produce de-identified results from the electronic medical records that fit the above criteria. Data prior to and after intervention were compared.

This study is exempt from the Institutional Review Board under guideline 45 CFR 46.102(d) in accordance with New York State Public Health Law 2805 j through m; and New York State Education Law 6527; and Federal Law 109-41.

## Results

Four providers were recruited during the 2017 to 2019 influenza seasons. Over 984 patients met inclusion criteria, with normal distribution of ages (18-80), race, and sex. The aforementioned interventions with provider and staff education were implemented between the 2018 and 2019 influenza season. The average vaccination rate prior to the intervention was 9.25-13.60%. The average vaccination rate after intervention 91.34% (Table 1).

**Table 1:** MSBI Allergy and Clinical Immunology Influenza Vaccination Rates by Provider Intervention occurred between the 2018 and 2019 influenza season.

Provider	Metric	2017 Season	2018 Season	2019 Season
Provider A	Immunization count	29	37	162
	Patient count	323	292	165
	Rate	8.98%	12.67%	98.18%
Provider B	Immunization count	4	7	17
	Patient count	34	26	25
	Rate	11.76%	26.92%	68.00%
Provider C	Immunization count	4	4	8
	Patient count	43	34	10
	Rate	9.30%	11.76%	80.00%
Provider D	Immunization count	N/A	0	24
	Patient count	N/A	1	31
	Rate	N/A	0.00%	77.42%
Total	Immunization count	37	48	211
	Patient count	400	353	231
	Rate	9.35%	13.60%	91.34%

## Discussion

On November 9, 2020, Pfizer and BioNTech announced the initial successful results of phase 3 studies on their messenger ribonucleic acid-based vaccine candidate, BNT162b2, in targeting the SARS-CoV-2 virus [8]. In the following weeks, Moderna, AstraZeneca, and other companies around the world have also begun finalizing results and starting steps toward seeking emergency approval [9]. Initial studies have shown a vaccine efficacy of 95.0% and 94.5%, for Pfizer and Moderna respectively, which greatly contrasts that of the influenza vaccine of 40-60% [1,8,9]. Prior to this, increased personal hygiene, use of protective equipment, and social distancing measures were the major public health strategies in controlling and mitigating the effects of the COVID-19 pandemic [13]. Since then, comparisons have been made to compare the effect and impact of COVID-19 to influenza pandemics of history, in which the development and utilization of influenza vaccines have decreased associated morbidity and mortality [14]. As noted in previous wide-scale vaccination attempts throughout history, shared decision making, and intensive education/outreach efforts are needed by physicians and patients alike to overcome vaccine hesitancy.

Defined as the delay in the acceptance or refusal of immunizations despite availability, the reasons for vaccine hesitancy and refusal are varied and mirror the experiential, racial, and socioeconomic diversity in the United States [15]. Common reasons identified include, but are not limited to risk perception of the disease or vaccine, beliefs of the vaccine efficacy, knowledge about the disease, or lack of recommendation from medical personnel [15]. As noted with newer vaccines such as Rotarix for rotavirus or frequently changing ones such as the influenza vaccine, misinformation in efficacy or adverse effects can create negative perceptions that cloud patient judgement and decision making [16,17]. With the advent of social and alternative media for information dissemination, anti-vaccination efforts have gained traction in the past decade, leading to the resurgence of childhood diseases [18]. Coupled with the recent politicization of public health efforts and scientific facts, similar hesitancy and resistance have been seen with potential COVID-19 vaccine efforts. Multi-level interventions that are tailored to address the specific concerns of the individual or community have been demonstrated to have the most effective outcome on vaccination rates [19]. As seen with the quality improvement assessment and intervention done at the aforementioned clinic, this approach was critical in increasing influenza vaccination rates.

Vaccine hesitancy can present in a spectrum, ranging from mild doubt to extreme distrust, which in turn affects the healthcare provider’s ability to counsel and encourage vaccination. Identification of the unique factors for each patient is important as this can create a personalized approach to address the specific concern. Risk perception, distrust in government, and suspicion of the healthcare system are among the most commonly cited reasons for influenza vaccine refusal [15]. With influenza, patients often believe that they are not at risk due to survivorship bias and the generally benign clinical course if infected [17]. The influenza disease burden is difficult for many patients to appreciate as in the past decade, out of the 45 million infected influenza patients in the United States, only a minute fraction required hospitalization or resulted in death (810,000 and 61,000, respectively, accounting for 1.8% and 0.14%) [20]. As higher morbidity and mortality for influenza are attributed to lower socioeconomic status or other social determinants of health, the general population can adopt cognitive biases when faced with obtaining a vaccine for public or preventive health measures [3]. Facing comparable challenges, public opinion of COVID-19 disease control protocols such as mask mandates, social distancing, shelter-in-place orders, and presumptive vaccination efforts, have been met with mixed results in adherence and support [13].

Vaccine efficacy is also a patient concern, unlike childhood immunizations such as polio and measles, the influenza virus can still infect those who receive the vaccination. This is largely due to the high mutation rate of the influenza virus and how the standard influenza vaccine is a quadrivalent formulation in which researchers attempt to predict the four most likely strains across over 100 hemagglutinin and neuraminidase subtype combinations [21]. According to the Centers for Disease Control and Prevention, influenza vaccine efficacy typically averages around 60% and can be less in years when new strains undergo antigenic drifts and epigenetic shifts [5]. This variability contributes to labile efficacy as seen in the low effectiveness of disease prevention in the 2014-2015 season and the complete ineffectiveness in the 2008-2009 season for the influenza A subtype H1NI pandemic [21]. By comparison, the measles vaccine achieved a 90% vaccine efficacy with only a single dose [5,22]. Despite promising news of greater than 90% efficacy, the fast-tracking of COVID-19 vaccines and further politicization of the pandemic will undoubtedly raise similar apprehension for patients and providers alike in the upcoming months.

Direct patient education is often the solution for healthcare providers as they attempt to balance beneficence and non-maleficence with respect for patient autonomy. Studies have shown, however, that direct education may increase vaccination reluctance as patients utilize refusal as a psychological defense reaction to perceived challenges of their beliefs and knowledge [23]. Several strategies have been implemented in vaccination efforts over the past decades with variable levels of patient engagement and success. Across diverse patient populations, motivational interviewing as a patient-centered communication style has been an effective method of improving vaccination rates as the conversation is centered around the patient and provides a respectful and non-judgmental setting to explore possible concerns [24]. An additional benefit of motivational interviewing is that it further enhances the patient-doctor relationship, as poor rapport/connection has been demonstrated to negatively influence vaccination choice [24]. As noted in research conducted during the early COVID-19 pandemic, the use of telehealth and infection control guidelines can stunt the humanistic connection that is typically required during patient education [25]. It is important for providers to recognize that vaccination beliefs are not static and can change from visit to visit.

Concurrent subcutaneous immunotherapy (SCIT) with vaccination and the presence of egg allergies are two concerns identified by allergy clinic providers as potential areas of concern for patients [26]. The influenza vaccine uses egg-based manufacturing processes to incubate the influenza virus, such that the typical ovalbumin content of less than 1 µg per 50-ml dose [27]. Historical recommendations previously cautioned patients and providers regarding influenza vaccination in egg-allergic patients. Anaphylaxis related to the influenza vaccine is rare, with data from the Vaccine Adverse Event Reporting System unable to correlate reactions to egg allergies [28]. According to the American Academy of Allergy, Asthma, and Immunology (AAAAI), influenza vaccines should be administered regardless of egg allergy severity and positive egg allergy is not a contraindication to vaccination [29]. Less research exists on concurrent SCIT administration with the influenza vaccine; however, the AAAAI and its counterparts across the world have released expert opinions regarding the safety and non-interaction of co-administration. With the MSBI providers, they were able to leverage their authority and expertise in the field of allergy and immunology to allay patient concerns and fears. As the COVID-19 vaccine becomes distributed, physicians will similarly need to leverage their positions and trust to encourage vaccination efforts.

While the strategies illustrated in this quality improvement research to improve influenza vaccination can be used in other public health campaigns and extrapolated to future COVID-19 vaccination efforts, there are limitations to this study. The most salient unknown is the rapidly evolving knowledge base and research for the SARS-CoV-2 virus. Compared to the decades of research that exists for influenza and other pathogens in the modern vaccination schedule, the COVID-19 vaccine has limited studies and an expedited timeline for safety and quality review [30]. Even the mechanism of action for the proposed vaccine is novel in using a messenger ribonucleic acid to produce an antigenic response instead of traditional viral proteins [8,9]. Another limitation is that this study focused primarily on the point of view of the provider by proxy of coding through the electronic medical record. Depending on if the encounter was coded correctly, this can result in a false representation of patient vaccine hesitancy (i.e., forgetting to input the procedural code for documentation of vaccine completion). Formalized surveys of providers and patients alike would also provide a better understanding around the challenges and motivations of influenza vaccine hesitancy and refusal. Ultimately, it is still unknown whether these motivations would be shared with the prospective COVID-19 vaccine.

## Conclusions

Due to the advent of social media and other external factors, the past two decades have presented a challenge for healthcare providers when recommending annual and routine vaccinations. As evidenced in the yearly battle with influenza and now the COVID-19 pandemic, it has become essential to identify and implement multi-level strategies to maximize vaccination rates, especially amid a global pandemic. Vaccine hesitancy, provider knowledge of adverse reactions, and accurate documentation were areas addressed in the aforementioned quality improvement project in improving influenza vaccination rates in an outpatient urban setting, with a focus on allergy and immunology providers. With COVID-19 vaccines reaching emergency approval stages, it is important for healthcare providers to start creating workflows and strategies to address patient inquiries. The importance of patient education and commitment in stopping the spread of the COVID-19 virus in their family and community can be leveraged with up to date and unbiased information in order to allow them to voice concerns and improve provider-patient trust through transparency. Continuous monitoring of vaccine rates is paramount to assess how these vaccines will affect social, economic, governmental, and global response to the pandemic in upcoming months. The influenza vaccination quality improvement project presented here can be used as a guideline for future evaluations of COVID-19 vaccination efforts.
